# Reduced antimicrobial consumption through enhanced pneumonia management in critically ill patients: outcomes of an antibiotic stewardship program in the intensive care unit

**DOI:** 10.3389/fmed.2025.1549355

**Published:** 2025-05-22

**Authors:** Asieb Sekandarzad, Annabelle Flügler, Anne Rheinboldt, David Rother, Gesche Först, Siegbert Rieg, Alexander Supady, Achim Lother, Dawid Leander Staudacher, Tobias Wengenmayer, Winfried V. Kern, Paul Marc Biever

**Affiliations:** ^1^Interdisciplinary Medical Intensive Care, Medical Center, Faculty of Medicine, University of Freiburg, Freiburg, Germany; ^2^Division of Infectious Diseases, Department of Medicine II, Medical Center, Faculty of Medicine, University of Freiburg, Freiburg, Germany

**Keywords:** antibiotic stewardship, intensive care unit (ICU), antimicrobial resistance pattern, reduction of antimicrobial consumption, intervention bundle, multifaceted approach

## Abstract

**Background:**

Critically ill patients in the intensive care unit (ICU) who are suspected of having pneumonia are frequently treated with broad-spectrum antimicrobials even when the diagnosis remains uncertain. While appropriate antimicrobial therapy offers undeniable benefits, its inappropriate or excessive use can lead to harmful side effects. This study examines the impact of an antimicrobial stewardship program (ASP) in the ICU on both diagnostic accuracy and antimicrobial consumption in critically ill patients with pneumonia.

**Methods:**

This cohort study compares a prospective cohort with matched controls from a retrospective sample in the ICU of a tertiary hospital. An ASP was implemented focusing on microbiological sampling of bacteria and antimicrobial therapy. Primary endpoint was days of therapy (DOTs). Secondary endpoints were number of respiratory samples (RS), identification of relevant bacteria in RS and diagnostic accuracy of pneumonia. Clinical safety outcome parameters were length of stay, length of invasive mechanical ventilation and ICU mortality until day 28.

**Results:**

A total of 200 patients were assigned to the intervention group (IG) and 200 to the control group (CG). The overall DOTs per patient were 12.95 [95% confidence interval (CI) 11.42 to 14.47] in the CG compared to 9.91 (CI 8.97 to 10.82) in the IG (*p* = 0.036), with no unfavorable findings in safety outcome parameters. DOTs for meropenem were 2.74 (CI 2.14 to 3.34) in the CG vs. 1.13 (CI 0.76 to 1.49) in the IG (*p* < 0.001), DOTs for piperacillin/tazobactam were 3.66 (CI 3.16 to 4.15) vs. 2.78 (CI 2.33 to 3.22; *p* = 0.011), and DOTs for ampicillin/sulbactam were 1.49 (CI 1.15 to 1.82) vs. 2.63 (CI 2.25 to 3.02; *p* < 0.001). Relevant bacteria in RS were detected more frequently in the IG, with *n* = 91 compared to *n* = 61 in the CG (*p* = 0.003).

**Conclusion:**

Implementation of an ASP in the ICU effectively reduces broad-spectrum antimicrobial consumption in critically ill patients with pneumonia without compromising patient safety.

## Background

Respiratory tract infections are frequent in the ICU (1, 2). These patients often receive broad-spectrum antimicrobials, even in the absence of confirmed bacterial infection. Mortality rates associated with pneumonia in the intensive care unit (ICU) remains high, which despite ongoing advancements in antimicrobial therapy (AMT), diagnostic tools, and evidence-based treatment guidelines, still ranges from 15 to 50% (3). This leads to overtreatment and increased antimicrobial consumption in critically ill patients. The choice of targeted AMT in the initial time course of the disease is crucial and challenging. Inappropriate AMT and uncritical use of broad-spectrum antimicrobials contribute to increased morbidity and mortality (4–8), escalating health care costs and growing antimicrobial resistance (9–16). Therefore, more precise and targeted use of antimicrobials is warranted. Antibiotic stewardship programs (ASP) have demonstrated to improve diagnostic procedures and treatments for various infectious diseases while at the same time leading to a reduction of antimicrobial use and deceleration of the development of antimicrobial resistance of pathogens (12, 17). It is desirable that ICU physicians develop a certain level of competence in antimicrobial stewardship principles (1, 2, 18). We implemented an ASP intervention bundle that combined multiple strategies based on established antibiotic stewardship methods (19–21). The aim of this study was to evaluate the effect of an ASP in the ICU on diagnostic accuracy and the use of broad-spectrum antimicrobial in critically ill patients with suspected pneumonia.

## Methods

### Study design, setting, and patient inclusion criteria

Antibiotic stewardship program in the Intensive care unit (ABSINT) examined the impact of an ASP for patients with pneumonia treated in the medical intensive care units (MICU) at the Freiburg University Medical Center, an academic tertiary referral center. The MICU consists of 28 beds, located on two spatially independent wards. ABSINT was a cohort study comparing a prospective cohort with matched controls from a retrospective sample treated on the same ICUs before initiation of the intervention. At baseline we introduced an ASP intervention bundle targeting at optimized treatment of patients with pneumonia. This intervention bundle included a checklist for a standardized diagnostic workup and a rational use of antimicrobials (Supplementary material 5). Patients eligible for study participation were adults aged 18 years or above admitted to the MICU and were to receive AMT for a suspected or confirmed diagnosis of pneumonia, as determined by the treating physician(s). The study cohort included patients with community acquired pneumonia (CAP), hospital acquired pneumonia (HAP) and ventilator associated pneumonia (VAP). Patients with a length of stay in the ICU of <72 h were excluded. The sample size was determined based on practicability and prior studies with similar methodologies (22, 23). Patients in both groups were selected randomly. During the 12-month prospective observation period 200 patients were enrolled—100 patients were treated on MICU 1 and 100 on MICU 2.

**Figure 1:**
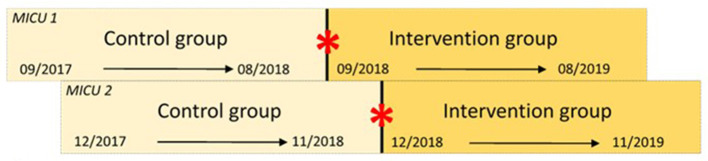
Schematic illustration of the different intervention timepoints on the different intervention timepoints on MICU 1 and MICU 2. For reasons of practicability, we first initiated the study on MICU 1 and after successful implementation we introduced the intervention on MICU 2 three months later. Thick bar in the middle marks the baseline and the red asterisk marks the intervention timepoint. Top row illustrates timeline of MICU 1 and bottom row timeline of MICU 2. MICU, medical intensive care unit.

The enrollment periods differed slightly between the two medical MICUs due to practical considerations (Figure 1). In MICU 1, patients in the control group were enrolled between September 2017 and August 2018, while in MICU 2, control group enrollment occurred from December 2017 to November 2018. The ASP intervention was initiated in MICU 1 from September 2018 to August 2019, and in MICU 2 from December 2018 to November 2019. We deliberately chose nearly identical timeframes for both the control and intervention periods across the MICUs to account for potential seasonal variations in respiratory tract infections, thereby reducing seasonal bias. Additionally the timeframe was set to 11 months to help balance out seasonal effects, particularly those related to the winter months. We selected 200 patients for the control group which were treated on the same MICUs before initiation of the ASP intervention by identification from a list based on International Statistical Classification of Diseases coded patients for pneumonia (ICD codes J12., J18., U69. 01) and matched to specific criterias (Supplementary material 2). The study was conducted in accordance with the Declaration of Helsinki. The study was approved by the Freiburg University Research Ethics Committee (EK-Freiburg 286/18) and the need for informed consent was waived. The study conformed to the STROBE (Strengthening the Reporting of Observational Studies in Epidemiology) reporting guidelines (24).

### Intervention

The ASP intervention comprised measures defined by the department's ASP team targeting at optimized management of patients with pneumonia. The ASP team consisted of three board certified intensivists and a clinical pharmacist. The intensivists assisted the other clinical team members in implementing the guideline. The clinical pharmacist standardized application modes and dosage of various antimicrobial agents. ICU physicians on duty were in full charge of prescribing anti-infectious treatments.

**Figure 2:**
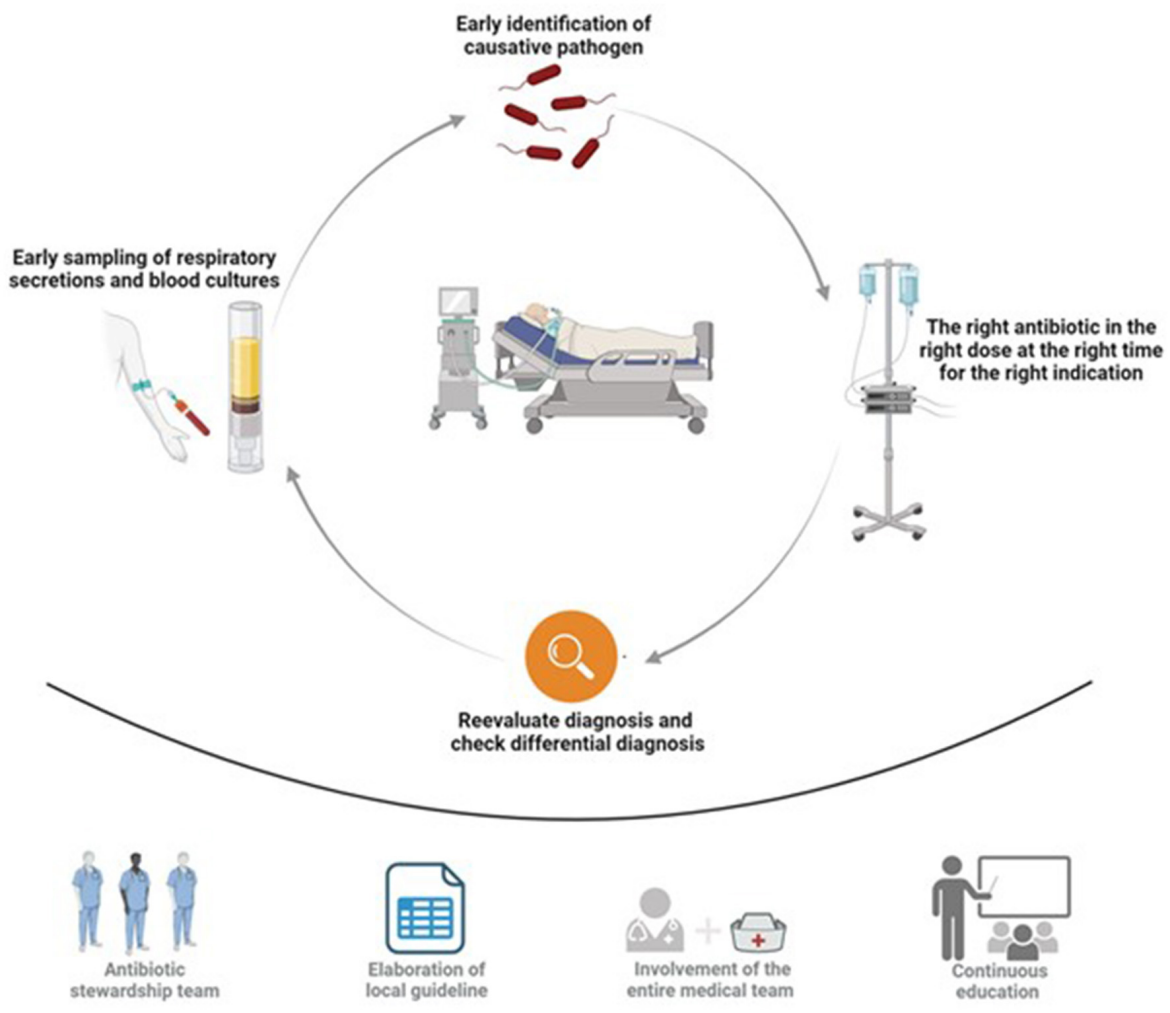
Graphical abstract of the intervention bundle. Top illustration visualizes the diagnostic core concept of the intervention while the bottom illustration demonstrates and emphasizes the multilevel approach of the intervention.

The key aspects of the intervention bundle basically comprised three components: Elaboration of the local guideline, education of the entire ICU medical team and patient consultation (Figure 2). The local ASP guideline (Supplementary material 6) considered current evidence-based national treatment guidelines and local antimicrobial resistance patterns. The guideline was easily accessible via the hospital's intranet and highlights specific AMT strategies for different entities of pneumonia and specifies diagnostic criteria for categorization of diagnostic certainty (pneumonia confirmed or pneumonia possible) were predefined (Supplementary materials 4, 6). Educational events included initial specific team briefings, summarizing the revised guidelines and explaining the overall strategy. Repeated educational rounds for physicians and nursing team were held in the first 3 months of implementation. Additionally, posters and checklists were available (Supplementary material 5). Feedback strategies such as survey tools (in written form and electronically) were applied. Intensified patient consultations with academic detailing and recommendation to enhance guideline adherence were mostly held during day time. Consultations on weekends and night shifts were exceptionally held if a team member of the ASP team was on duty.

### Standard procedures in the control group

Medical management of patients with pneumonia in the control group was at the discretion of the ICU physicians considering current evidence based on national and international treatment guidelines for the management of community and hospital acquired pneumonia (25, 26). Neither a specific local guideline, nor a microbiological standard was available at the time for the management of pneumonia in the ICU. An ICU-specific antimicrobial stewardship team was not present. In case of complex infections, an infectious diseases specialist was consulted. The use of broader and longer antimicrobial therapies, often conducted with piperacillin/tazobactam or meropenem was not specifically controlled or restricted. Cephalosporins and fluoroquinolones were only rarely used first-line, a continuing result of a previous ASP in the MICU in 2012 (27).

### Primary and secondary endpoints

The primary endpoint of ABSINT was “days of therapy” (DOTs) until day 28 or ICU discharge. DOTs describe the sum of days with any amount of a specific antimicrobial agent administered to an individual patient. For example, AMT with ampicillin/sulbactam for 6 days while receiving clarithromycin for 3 days, is reported as 9 DOTs. Only antimicrobial agents were taken into account, antifungal and antiviral agents were not investigated. DOTs were calculated for overall antimicrobial agent use as well as specifically for ampicillin/sulbactam, piperacillin/tazobactam and meropenem. Secondary endpoints were number of respiratory samples, identification of typical bacteria in respiratory secretions, diagnostic reliability of the diagnosis of pneumonia according to predefined criteria in our local guideline. Safety clinical outcome parameters were ICU mortality, length of ICU stay (LOS) and time of invasive mechanical ventilation (IMV) until day 28.

### Data collection, definitions and statistical analysis

Data were systematically collected from the electronic patient data management system. Patients were defined “AMT naive” if no AMT was administered within 72 h prior to study enrollment. Detection of certain bacteria were regarded as relevant in respiratory secretions (Supplementary material 4). Categorical and continuous data were presented as numbers and/or percentages, means, standard deviation, as appropriate. Categorical variables were compared using Fisher's exact test. Quantitative continuous variables were compared using the Mann-Whitney U test. *P* ≤ 0.05 was considered statistically significant. To mitigate differences between the two groups, a propensity score matching was conducted using a nearest neighbor matching algorithm with a caliper of 0.05. Matching was performed for age, admission via emergency department, SOFA score, invasive mechanical ventilation on admission, immunsuppression, status post cardiac arrest and no antimicrobial exposure 3 days before enrollment. Statistical calculations were conducted using IBM SPSS statistics 25.0 (Armonk, NY: IBM Corp, 2017) and GraphPad Prism Version 10.1.0 for Windows (GraphPad Software, Boston, Massachusetts USA). For figures GraphPad Prism Version 10.1.0 was employed. The graphical abstract was designed using BioRender.com.

## Results

### Baseline characteristics

Four hundred patients were enrolled, with 200 assigned to control and intervention groups, respectively. 239 of 400 patients (60%) received invasive mechanical ventilation, 63 patients (16%) suffered from septic shock. Primary reasons for admission to the ICU were pneumonia in 112 patients (30%), cardiac arrest in 108 patients (27%) or other respiratory insufficiency in 67 patients (17%). Patients in the control group had less often received no prior AMT (75/200 vs. 107/200; *p* = 0.003) compared to the intervention group but presented more frequently with HAP (88/200 vs. 62/200; *p* = 0.01). Admissions via the emergency room and cardiac arrest were less common in the control group (73/200 vs. 103/200; *p* = 0.003 and 42/200 vs. 66/200; *p* = 0.009 respectively). Proven influenza (by PCR) and non-invasive ventilation mode were more frequently present in the control group (28/200 vs. 9/200; *p* = 0.002 and 41/200 vs. 23/200; *p* = 0.019). The remaining baseline characteristics did not differ significantly between both groups (Table 1).

**Table 1:** Comparison of baseline characteristics between the control and intervention groups.

**Patients' characteristics**	**All (*n* = 400)**	**Control group (*n* = 200)**	**Intervention group (*n* = 200)**	**p-value**
Age	64.7 (15)	64.5 (15)	64.9 (15)	0.992^†^
Male	263 (67)	132 (66)	131 (66)	1.0
Comorbidities^*^	355 (89)	178 (89)	177 (89)	1.0
- Cardiac	272	140 (70)	132 (66)	0.453
- Pulmonary	122	60 (30)	62 (31)	0.913
- Of which COPD	60	26 (13)	34 (17)	0.327
- Hepatic	47	24 (12)	23 (12)	1.0
- Renal	74	30 (15)	44 (22)	0.093
- Neurologic/psychiatric	60	26 (13)	34 (17)	0.327
- Hemato-oncologic	61	33 (17)	28 (14)	0.578
Immunosuppression	62 (16)	31 (16)	31 (16)	1.0
Colonization MRSA	5 (1)	3 (2)	2 (1)	1.0
Colonization multidrug resistant gram negative bacteria	31 (8)	17 (9)	14 (7)	0.709
Risk of multidrug resistant gram negative bacteria	58 (15)	29 (15)	29 (15)	1.0
Risk of Pseudomonas aeruginosa^**^	31 (8)	15 (8)	16 (8)	1.0
**Admisssion path**				
Emergency department	176 (44)	73 (37)	103 (52)	0.003
Transfer from lower level hospital	123 (31)	68 (34)	55 (28)	0.193
Inpatient from other departments	101 (26)	59 (30)	42 (21)	0.065
**Medical reason for primary admission on ICU**				
Pneumonia	112 (30)	56 (28)	56 (28)	1.0
Cardiac arrest	108 (27)	42 (21)	66 (33)	0.009
Other cardiac presentations	53 (13)	33 (17)	20 (10)	0.076
Other respiratory insufficiency	67 (17)	38 (19)	29 (15)	0.284
Other	60 (15)	31 (16)	29 (15)	0.888
SOFA score	10.0 (5)	9.8 (5)	10.1 (4)	0.435^†^
Septic shock	63 (16)	33 (17)	30 (15)	0.783
CAP	250 (63)	112 (56)	138 (69)	0.01
HAP	150 (38)	88 (44)	62 (31)	0.01
Influenza A/B (PCR positive)	37 (9)	28 (14)	9 (4.5)	0.002
**Oxygen support**				
No oxygen support	2 (1)	1 (1)	1 (1)	1.0
Supplementary oxygen	69 (17)	33 (17)	36 (18)	0.791
Nasal high-flow	26 (7)	10 (5)	16 (8)	0.310
Non-invasive ventilation	64 (16)	41 (21)	23 (12)	0.019
Invasive mechanical ventilation	239 (60)	115 (58)	124 (62)	0.415
Of whom VV or VA ECMO	43 (11)	20 (10)	23 (12)	0.747
No prior antimicrobial therapy within 3 day before enrolment	182 (46)	75 (38)	107 (54)	0.003

Values are mean (standard deviation) or number (%). If not marked otherwise statistical tests were performed with Fisher's exact test.

† Mann-Whitney U test was performed.

* Multiple comorbidities possible.

** Risk of *Pseudomonas aeruginosa* was defined as presence of COPD GOLD IV or cystic fibrosis or bronchiectasis or known colonization with *Pseudomonas aeruginosa*.

MRSA, methicilin resistent staphylococcus aureus; SOFA, sequential organ failure assessment; VV, veno-venous; VA, veno-arterial; ECMO, extracorporeal membrane oxygenation; CAP, community acquired pneumonia; HAP, hospital acquired pneumonia; VAP, ventilator associated pneumonia.

### Days of therapy

**Figure 3:**
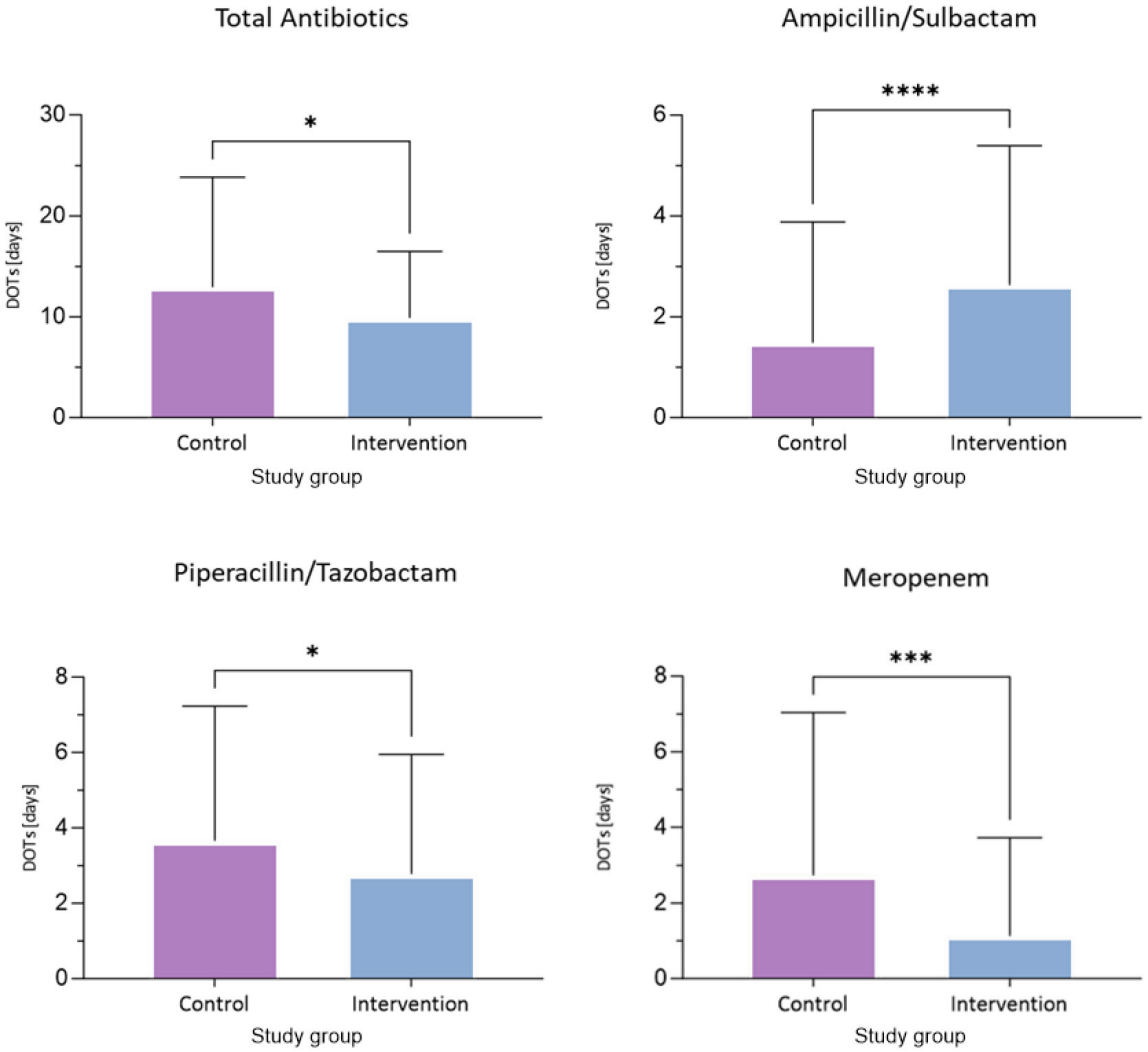
Comparison of days of antimicrobial therapy (DOTs) per patient between the control and intervention groups. **Top row:** presentation of DOTs of total antimicrobials and ampicillin/sulbactam in the control- and intervention group. **Bottom row:** presentation of dots of piperacillin/tazobactam and meropenem, between both groups respectively. Bar represents mean with standard deviation. *p ≤ 0.05, ***p ≤ 0.001, ****P ≤ 0.00001.

The overall DOTs per patient in the control group were 12.95 (SD 10.9) and 9.91 (SD 6.6) in the intervention group resulting in a significant decrease by 23.6% (*p* = 0.036; Figure 3, Supplementary material 1). The DOTs for ampicillin/sulbactam in the control group were 1.49 (SD 2.4) vs. 2.63 (SD 2.8) in the intervention group showing an increase by 77.1% (*p* < 0.001). The DOTs for piperacillin/tazobactam were 3.66 (SD 3.6) in the control group vs. 2.78 (SD 3.2) in the intervention group, thereby demonstrating a decrease by 24.1% (*p* = 0.011). The DOTs for meropenem in the control group were 2.74 (SD 4.3) vs. 1.13 (SD 2.6) in the intervention group, reflecting a significant reduction of 58.9% (*p* < 0.001).

### Secondary endpoints

In the control group at least one respiratory tract sample was taken from 115 out of 200 patients (58%) whereas in the intervention group at least one sample from the respiratory tract was taken from 158 of 200 patients (79%), showing a significant difference (*p* < 0.001; Table 2). Tracheal secretions were the most frequent type of respiratory tract samples in both groups, with a significant increase in the intervention group (82/200 vs. 143/200; *p* < 0.001). The number of patients in whom at least one relevant bacterial species could be identified was higher in the intervention group (61/200 vs. 91/200, *p* = 0.003). The microbiological spectrum in both groups showed no significant difference (Supplementary material 3).

**Table 2:** Comparison of respiratory sampling and findings of relevant bacterial pathogen between the control and intervention groups.

**Patients**	**All (*n* = 400)**	**Control group (*n* = 200)**	**Intervention group (*n* = 200)**	***p*-value**
Number of patients with respiratory sample (%)	*n* = 273 (68)	*n* = 115 (58)	*n* = 158 (79)	<0.001
**Number of types of respiratory sample per patient**				
BAL/bronchial secretions	65	43	22	0.006
Tracheal secretions	225	82	143	<0.001
Number of patients with at least one relevant bacterial pathogen in respiratory sample	*n* = 152 (38)	*n* = 61 (31)	*n* = 91 (46)	0.003
**Subgroup without prior antimicrobial therapy**				
Number of patients	*n* = 182	*n* = 75	*n* = 107	0.002
Number of patients with at least one relevant bacterial pathogen	82 (45)	22 (29)	60 (56)	0.001

Values expressed as numbers and/or percent in brackets. Statistical tests were performed with Fishers's exact test. BAL, bronchoalveolar lavage.

**Figure 4:**
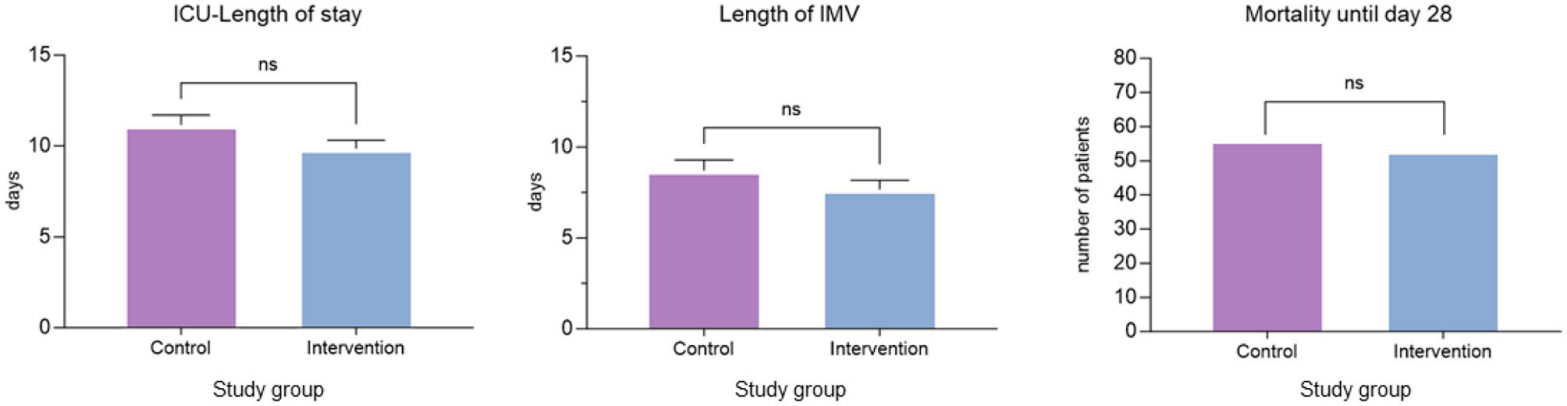
Safety endpoints. Comparison of ICU-length of stay, length of IMV and mortality until days 28 between the control and intervention groups. Bar represents means with standard deviation. IMV, invasive mechanical ventilation; ns, not significant.

The safety clinical outcome parameters ICU-LOS, length of IMV and mortality in ICU until day 28 were not significantly different between both groups. ICU-LOS was 11.16 (SD 7.7) in the control group and 9.85 (SD 6.6) in the intervention group, length of IMV was 8.70 (SD 8.3) in the control group and 7.66 (SD 7.2) in the intervention group and mortality in ICU until day 28 was not significantly different between both groups (56/200 vs. 53/200; Figure 4, Supplementary material 1).

### Diagnostic criteria of pneumonia

**Figure 5:**
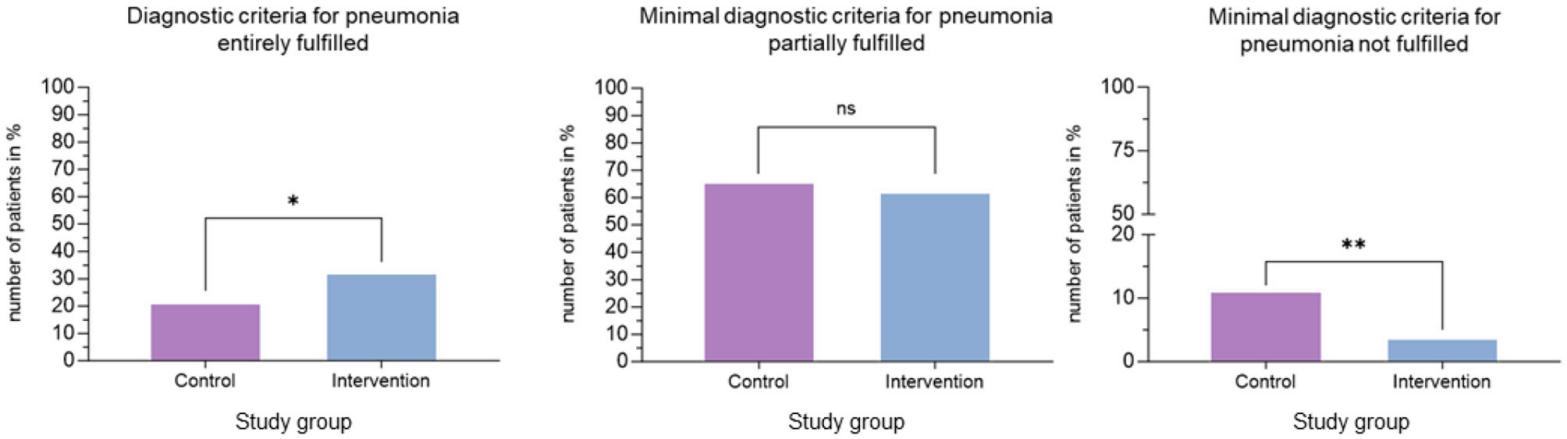
Diagnostic certainty. Assessing the percentages of patients in the control and intervention groups who met the diagnostic criteria of pneumonia according to the local guideline. The left chart shows number of patients in percent who entirely fulfilled diagnostic criteria for pneumonia. The middle chart displays number of patients (%) who did not meet minimal diagnostic criteria. If not marked statistical tests were performed with Fisher's exact test. *p ≤ 0.05, **p ≤ 0.01, n.s., not significant.

The proportion of patients who fully met the diagnostic criteria for pneumonia according to the local guideline was significantly higher in the intervention group (44/200 (22%) vs. 66/200 (33%); *p* = 0.019; Figure 5, Supplementary material 3). The number of patients who partially met the diagnostic criteria for pneumonia did not significantly differ between both groups [133/200 (67%) vs. 126/200 (63%); *p* = n.s.], whereas the number of patients who did not meet the predefined minimal diagnostic criteria for pneumonia was lower in the intervention group [23/200 (12%) vs. 8/200 (4%); *p* = 0.008].

### Propensity score matched cohort

To balance for differences in baseline characteristics we performed a propensity score matched analysis of 368 patients (*n* = 168 control, *n* = 200 intervention group). In this analysis the overall DOTs in the control group was 12.28 (SD 10.5) and 9.91 (SD 6.6) in the intervention group, indicating a decrease of 23.5%, which was not significant (*p* = 0.22). The DOTs for ampicillin/sulbactam were 1.64 (SD 2.5) in the control group and 2.63 (SD 2.8) in the intervention group showing a significant increase of 61.3% (*p* < 0.001). The DOTs for piperacillin/tazobactam were 3.63 (SD 3.6) in the control group and 2.78 (SD 3.2) in the intervention group demonstrating a significant reduction of 23.4% (*p* = 0.024). The DOTs for meropenem were 2.55 (SD 4.3) in the control group and 1.13 (SD 2.6) in the intervention group, resulting in a decrease of 55.8% (*p* = 0.002; Supplementary material 2). The safety clinical outcome parameters LOS (10.89 days, SD 7.4; vs. 9.85 days; SD 6.5; *p* = 0.33), length of IMV (8.49 days; SD 8.1 vs. 7.66 days; SD 7.2; *p* = 0.57) and mortality until day 28 [*n* = 45 (26.8%) vs. n = 53 (26.5%); *p* = 0.951] were not statistically different between control and intervention group (Supplementary material 2).

## Discussion

This study showed a significant modification of AMT for the treatment of critically ill patients with pneumonia in the MICU by the implementation of an intervention bundle within an ASP, resulting in shortened treatment and reduced broad-spectrum betalactam use. In addition to a significant reduction of piperacillin/tazobactam and meropenem use, an increase of ampicillin/sulbactam was observed in the intervention group. This modification of antimicrobial use is explainable by a strong adherence to the local guideline including a meticulous diagnostic approach, which was intended by the ASP and enabled a more frequent appropriate pathogen identification. The reduction of the use of piperacillin/tazobactam and especially of meropenem, which still represents a reserve antimicrobial, can be explained by its restrictive indication based on predefined risk factors for multidrug resistant bacteria. In this study the incidence of multidrug resistant gram-negative bacteria was surprisingly low (Supplementary material 3) which reinforces the need and importance to know the local epidemiology and bacterial pathogen resistance patterns. Subsequently, our institution restrictively prescribes carbapenems only for patients with known colonization by multidrug resistant bacteria within the past 3 months and not for patients with other theoretical risk factors for the presence of multidrug resistant bacteria. However this rather moderate rate for multidrug resistant bacteria in southern Germany (28) does not imply an universal applicable therapeutic implication. ICU physicians often argue that in the threat to life and limb the choice of a penicillin with more narrow spectrum puts life in danger and increases mortality. From an ASP point of view it is not advisable to define reductions of quantitative antimicrobial use without assessing patient outcomes (29). Though ASP should try to evaluate an effect on clinical outcomes it is not its primary aim (29). A combined aim of reducing use of antimicrobials (DOTs) with neutral effects on patient outcomes (safety clinical parameters) was our realistic approach. The current literature regarding ASP concepts says that ASPs could have an effect on a variety of outcomes. Yet, currently no consensus on best practice exists (29). In this study, the safety clinical outcome parameters (ICU-LOS, IMV and mortality until day 28) did not show detrimental effects between both groups.

Our results are in line with previous studies that have shown that de-escalation from broad-spectrum AMT in patients with ventilator associated pneumonia is possible without adverse effects in terms of survival (22, 30–32). To date most of the published data concerning ASP initiatives for critically ill patients with pneumonia mainly focused on specific clinical scenarios such as procalcitonin guided AMT duration or use of multiplex polymerase chain reaction for detecting bacteria in respiratory samples (33, 34). However, these studies have not uniformly demonstrated that antimicrobial consumption was reducible through an intervention. ASPs like the one presented here, which take a multidimensional approach to the comprehensive care management of critically ill pneumonia patients, are rare, likely due to the complexity of implementation and evaluation. Interestingly, a recently published ASP targeting only diagnostic optimization in patients with suspicion of VAP could show a significant reduction in broad-spectrum antimicrobial use which emphasizes the key role of adequate diagnostics (35). The diagnostic approach, as presented in this study, embodied the backbone of the ASP and enabled a reduced and focused use of antimicrobials. Indeed, we were able to demonstrate a significant increase in the identification of relevant bacteria, allowing a targeted AMT. Moreover, strict reconsideration of differential diagnosis within 3 days after starting the empiric therapy has to be considered as essential. It is the combination of synergistic strategies that geared toward a better diagnostic certainty, as observed in this study. More patients in the intervention group entirely met predefined diagnostic criteria of pneumonia while fewer patients didn't even meet criteria of possible pneumonia. However, one has to recognize that the accurate diagnosis of pneumonia, especially in critically ill patients, is very challenging due to the lack of a gold standard for the precise diagnosis of pneumonia (36). In order to preserve the success and sustainability of this intervention its core concept was integrated into the clinical routine after the study was completed (29). Any ASP has higher chances of long-term success if persistent and continuous education is applied, all stakeholders (physicians, pharmacists and nursing staff) are involved and a dedicated team takes care of the program. Otherwise a relapse of earlier behavior will occur because old habits die hard (37–42). The MICU serves as an educational institution addressing medical students, residents and fellows who hopefully will adapt ASP concepts and evolve as ASP multipliers during their continuous medical careers. The indiscriminate use of broad-spectrum antimicrobials has significantly contributed to the evolution of antimicrobial resistance, it poses a serious threat to healthcare systems worldwide (43). In fact, broad-spectrum antimicrobials, when not really indicated, may increase mortality (44). Since large proportions of any hospital‘s use of parenteral antimicrobials occur in the ICU it is explicitly recommended that ASP should be regarded as a core competency of critical care physicians (9, 10, 14, 45, 46). Our study has shown rationale antimicrobial therapy in the setting of an implemented ASP is feasible and successful. This study has several limitations. First of all the studies design is not equivalent to a prospective randomized study and holds the potential of introducing selection or assignment bias. Indeed the number of patients who did not receive AMT prior to enrollment in ABSINT and the number of patients with cardiac arrest or CAPs were higher in the intervention group, whereas the number of patients with influenza pneumonia were fewer in the intervention group. These aspects were addressed by propensity score matching analysis. We demonstrated a relative reduction of the overall DOTs by 23% in the propensity score cohort, comparable to the reduction in the unmatched cohort which did not reach statistical significance. By reducing the sample size due to matching, statistical power might be limited, making it harder to detect a significant effect. Furthermore, propensity score-matching attempts to balance measured confounders while important unmeasured confounders could remain unaccounted and therefore could still influence the result. Though the *p*-value is not significant, we think that the observed effect size may still be clinically meaningful. We acknowledge the higher proportion of HAPs in the control group. This might have resulted in different prescription of antipseudomal beta-lactams. This aspect may have impacted the observed differences in treatment patterns. All patients without any AMT for suspected pneumonia have been excluded, so it remains uncertain to what extent there might be a further reduction in DOTs. A prospective parallel control group to mitigate temporal bias is lacking. Exclusion of patients who stayed on ICU for <72 h and lack of post-ICU follow-up needs to be considered as potential confounders.

## Conclusion

Implementation of an antimicrobial stewardship program within the ICU was feasible and effective in reducing the use of broad-spectrum antimicrobials among critically ill patients with pneumonia, without negatively impacting key clinical outcomes, including ICU mortality, length of stay in the ICU, and duration of mechanical ventilation.

## Data Availability

The raw data supporting the conclusions of this article will be made available by the authors, without undue reservation.
