# Supplementary material for: Reduced antimicrobial consumption through enhanced pneumonia management in critically ill patients: outcomes of an antibiotic stewardship program in the intensive care unit
Source: Front Med (Lausanne). 2025 May 22;12:1549355. doi: 10.3389/fmed.2025.1549355 (PMC12139207; doi:10.3389/fmed.2025.1549355)
Supplement: Supplementary file 1 [file Table_1.docx]

# Supplement

# Overall population

Table 3. Comparison of days of therapy (DOTs) per patient in the control- and intervention group.

| **DOTs** | **All**  **(n = 368)** | **Control**  **group (n = 200)** | **Intervention**  **group (n = 200)** | **Relativ per cent change** | **p Value** |
| --- | --- | --- | --- | --- | --- |
| Overall | 11.43 ± 9.1 | 12.95 ± 10.9  (11.42; 14.47) | 9.91 ± 6.6  (8.97; 10.82) | -24% | **0.036** |
| Ampicillin/Sulbactam | 2.06 ± 2.6 | 1.49 ± 2.4  (1.15;1.82) | 2.63 ± 2.8  (2.25; 3.02) | +77% | **<0.001** |
| Piperacillin/Tazobactam | 3.22 ± 3.4 | 3.66 ± 3.6  (3.16;4.15) | 2.78 ± 3.2  (2.33;3.22) | -24% | **0.011** |
| Meropenem | 1.93 ± 3.6 | 2.74 ± 4.3  (2.14;3.34) | 1.13 ± 2.6  (0.76;1.49) | -59% | **<0.001** |

## Values expressed as mean ± standard deviation. 95% confidence intervals (CI) are shown in brackets.

## If not marked otherwise Mann-Whitney U test was performed.

Table 4. Comparison of length of stay (LOS), duration of invasive mechanical ventilation (IMV) and mortality in ICU in the control and intervention group.

|  | **All**  **( n = 400)** | **Control**  **group (n = 200)** | **Intervention**  **group (n = 200)** | **p Value** |
| --- | --- | --- | --- | --- |
| LOS until day 28 [days] | 10.51 ± 7.2 | 11.16 ± 7.7 | 9.85 ± 6.6 | 0.229 |
| Length of IMV until day 28 [days] | 8.180 ± 7.8 | 8.70 ± 8.3 | 7.66 ± 7.2 | 0.456 |
| Mortality in ICU until day 28 [days] | 109 (27) | 56 (28) | 53 (26.5) | 0.737 |

Values expressed as mean ± standard deviation or number (percent). Mann-Whitney U test was performed.

# Propensity score matched population

Table 5. Comparison of days of therapy (DOTs) per patient in the propensity score matched population of the control- and intervention group. Matching was performed for age, admission via emergency department, SOFA score, invasive mechanical ventilation on admission, immunsuppression, status post cardiac arrest and no antimicrobial exposure three days before enrolment.

| **DOTs** | **All**  **(n = 368)** | **Control**  **group (n = 168)** | **Intervention**  **Group (n = 200)** | **Relativ per cent change** | **p Value** |
| --- | --- | --- | --- | --- | --- |
| Overall | 10.99 ± 8.7 | 12.28 ± 10.5 | 9.91 ± 6.6 | -23.5% | 0.217 |
| Ampicillin/Sulbactam | 2.18 ± 2.7 | 1.64 ± 2.5 | 2.63 ± 2.8 | +61.3% | < 0.001 |
| Piperacillin/Tazobactam | 3.16 ± 3.4 | 3.63 ± 3.6 | 2.78 ± 3.2 | -23.4% | 0.024 |
| Meropenem | 1.77 ± 3.5 | 2.55 ± 4.3 | 1.13 ± 2.6 | -55.8% | 0.002 |

Values expressed as mean ± standard deviation or number (%). Matching was performed for age, admission via emergency department, SOFA score, invasive mechanical ventilation on admission, immunsuppression, status post cardiac arrest and no antimicrobial exposure three days before enrolment.

The matching procedure (Unmatched vs. Matched) resulted in substantial improvement in covariate balance. Admission via emergency, status post cardiac arrest and no prior antimicrobial therapy remained slightly above the threshold of 0.1 after matching (Standard Mean Deviations/SMDs 0.10-0.14). Sofa score und IMV on admission showed excellent balance post matching (SMD < 0.05), immunosuppression remained well balanced throughout and age shifted slightly beyond the 0.1 threshold and remains acceptable and symmetric.

Table 6. Comparison of LOS, duration of IMV and mortality on ICU in the propensity-score matched population of the control and intervention group.

|  | **All**  **(n = 400)** | **Control**  **group (n = 168)** | **Intervention**  **group (n = 200)** | **p Value** |
| --- | --- | --- | --- | --- |
| LOS until day 28* [days] | 10.33 ± 6.97 | 10.89 ± 7.43 | 9.85 ± 6.53 | 0.330 |
| Length of IMV until day 28 [days] | 8.04 ± 7.62 | 8.49 ± 8.09 | 7.66 ± 7.21 | 0.570 |
| Mortality until day 28 | 98 (26.6) | 45 (26.8) | 53 (26.5) | 0.951 |

Values expressed as mean ± standard error of the mean or number (percent). Mann-Whitney U test was performed.

Figure 6. Comparison of days of antimicrobial therapy (DOTs) per patient between the control and intervention group in the propensity score matched cohort. Control group (n = 168), intervention group (n = 200), any point represents a DOT, midline represents the mean and error bar represents the standard deviation.

# Additional findings

Table 7. Comparing fulfilment response of diagnostic criteria of pneumonia according to the local guideline between the control and intervention group

| Diagnosis criteria for pneumonia according to local guideline | | | | |
| --- | --- | --- | --- | --- |
|  | All  (n = 400) | Control  group (n = 200) | Intervention group (n = 200) | p Value |
| Diagnostic for pneumonia entirely fulfilled | 110 | 44 (22%) | 66 (33%) | **0.019** |
| Diagnostic criteria for pneumonia partially fulfilled | 259 | 133 (66.5%) | 126 (63%) | 0.53 |
| Diagnostic criteria for pneumonia not fulfilled | 31 | 23 (11.5%) | 8 (4%) | **0.008** |

Values expressed as numbers (%). Statistical tests were performed with Fisher`s exact test.

Table 8. Identification of demonstrated bacterial species in respiratory samples

| Spectrum of relevant bacterial species in respiratory samples | | | | |
| --- | --- | --- | --- | --- |
|  | All  (n = 400) | Control  group (n = 200) | Intervention group (n = 200) | p Value |
| Streptococcus pneumoniae | 13 | 4 | 9 | 0.258 |
| Staphylococcus aureus   - MRSA**^1^* | 41  2 | 14  2 | 27  0 | 0.046 |
| Enterbacterales   - Klebsiella spp. - E. coli - Serratia spp. - Enterobacter spp. - Other***^2^** | 25  20  9  12  16 | 8  6  3  5  9 | 17  14  6  7  7 | 0.096  0.106  0.502  0.771  0.799 |
| - Multidrug resistant gram negative bacteria | 3 | 2 | 1 | 1.0 |
| Pseudomonas aeruginosa   - Multidrug resistant | 10  2 | 2  1 | 8  1 | 0.105  1.0 |
| Haemophilus influenzae | 20 | 7 | 13 | 0.250 |
| Legionella pneumoniae | 3 | 1 | 2 | 1.0 |
| Other*^3^ | 13 | 3 | 10 | 0.087 |

Each species was counted once per patient. If not marked otherwise statistical tests were performed with Fisher`s exact test. **^1^MRSA* multi resistant staphylococcus aureus *^2^Citrobacter spp., Proteus spp., Morganella spp. *^3^Mycoplasma pneumoniae, PJP, Acinetobaccter baumanii, Moraxella catarrhalis.

Table 9. Comparison of days of therapy of given antimicrobial agents between control and intervention group

| Antimicrobial agents | Control | Intervention |
| --- | --- | --- |
| Ampicillin | 11 | 18 |
| Ampicillin/Sulbactam | 297 | 526 |
| Amoxicillin/Clavulansäure | 0 | 0 |
| Azithromycin | 11 | 2 |
| Cefazolin | 0 | 0 |
| Ceftazidim | 9 | 7 |
| Ceftazidim/Avibactam | 14 | 0 |
| Ceftriaxon | 53 | 97 |
| Cefuroxim | 7 | 2 |
| Ciprofloxacin | 49 | 20 |
| Clarithromycin | 204 | 197 |
| Clindamycin | 36 | 4 |
| Daptomycin | 0 | 0 |
| Doxycyclin | 1 | 0 |
| Flucloxacillin | 85 | 12 |
| Fosfomycin | 5 | 0 |
| Gentamicin | 25 | 0 |
| Imipenem | 12 | 4 |
| Levofloxacin | 41 | 27 |
| Linezolid | 55 | 51 |
| Meropenem | 547 | 225 |
| Metronidazol | 46 | 15 |
| Moxifloxacin | 10 | 4 |
| Norfloxacin | 0 | 1 |
| Penicillin G | 34 | 62 |
| Piperacillin/Tazobactam | 731 | 555 |
| Roxithromycin | 0 | 3 |
| Rifampicin | 57 | 0 |
| Tigecyclin | 25 | 0 |
| Tobramycin | 30 | 23 |
| Tobramycin inhalativ | 6 | 0 |
| Trimethroprim/Sulfamethoxazol | 49 | 56 |
| Vancomycin | 139 | 70 |

# Key aspects of the intervention bundle and the local guideline

Key aspects of the intervention bundle were addressed using a multifaceted approach. Established ASP tools - such as checklists, posters, surveys, educational activities (for the entire medical team) were applied to improve guideline adherence and formed the cornerstone of the bundle. The guideline was developed in collaboration with the Infectious Diseases department, taking into consideration the local antimicrobial resistance pattern and epidemiology. Implementation was led by the ABSINT team. Educational presentation of the local guideline was mandatory for all internal residents in the ICU. Additionally the nursing staff of the ICU was repetitively educated concerning the acquisition and importance of respiratory secretions and blood culture sampling, which should take place at the onset of infection and before start of antimicrobial therapy. The ABSINT team supported the entire medical staff in the ICU to employ our local guideline adequately. Consultations, by the ABSINT team were performed regularly. Key aspects of the local guideline were:

Criteria for diagnosis of pneumonia were defined as following:

1. Diagnosis of pneumonia was considered definite if typical lung infiltration (preferentially computertomography of the thorax or chest x-ray) and at least two of the following criteria were present: purulent respiratory secretion, leucocytosis and/or fever.
2. Diagnosis of pneumonia was considered possible if typical lung infiltration was questionable and at least two of the following criteria were present: leucocytosis, fever, purulent respiratory secretion, increasing oxygenation disorder and/or evidence of typical bacterias in RS *or* if no typical lung infiltration was confirmed but at least three of the following criteria were present: leucocytosis, fever, purulent respiratory secretion, increasing oxygenation disorder, known colonization with typical bacterias and/or any questionable lung infiltration.

- Sampling of respiratory secretions (preferably tracheal secretions) should rigorously be taken in patients (endotracheally intubated and non-intubated) before start of antimicrobial therapy, precisely in any case of suspected or possible pneumonia. Bronchoalveolar lavage (BAL) was not routinely applied but emphasized for selected cases e.g. for patients with severe immunosuppression or patients without clinical improvement under empiric antimicrobial therapy.
- Detection of the following bacteria were regarded as relevant in respiratory secretions: Staphylococcus aureus, Streptococcus pneumoniae, Escherichia coli, Klebsiella pneumoniae, Pseudomonas aeruginosa, Proteus mirabilis, Enterobacter cloacae, Morganella spp., Haemophilus influenzae, Citrobacter freundii, Serratia marcescens, Legionella pneumonia, Mycoplasma pneumoniae, Pneumocystis jirovecii, Acinetobaccter baumanii or Moraxella catarrhalis.
- Initial antimicrobial therapy needs to be determined considering the clinical situation and the risk of resistant bacteria in order to enable an individualized antimicrobial therapy to avoid undertreatment.
- Rather restrictive definition of patients at risk with resistant bacteria: presence of known colonization with resistant bacterias or history of extensive broad spectrum antimicrobial therapy in the last 3 months or end stage chronic obstructive pulmonary disease; no general suspicion of patients at risk with resistant bacterias was suspected e.g. in patients with liver disease or renal replacement therapy.
- Recommendation for ampicillin/sulbactam for CAP and piperacillin/tazobactam for HAP empirically, in case of no risk of or presence of resistant bacteriass. Additional recommendation for clarithromycin and tobramycin for severe CAP or septic shock, respectively.
- Recommendation for carbapenems empirically only in exceptional cases with presence of high risk or proof of antimicrobial resistance pattern against piperacillin/tazobactam and especially in presence of septic shock.
- Duration of antimicrobial therapy: 5 days for not severe pneumonia (lack of oxygen supplementation) and 7 days for severe pneumonia (requiring oxygen supplementation), prolonged therapy only in exceptional cases.
- Daily evaluation of antimicrobial agent and dose for the given causative bacterias.
- Continuously and critically evaluation of infectious vs. non-infectious diagnosis.
- Dismiss antimicrobial therapy courageously and early enough if diagnosis other than bacterial pneumonia is more probable.

# Checklist


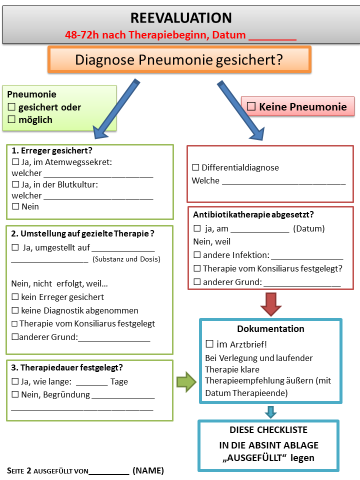

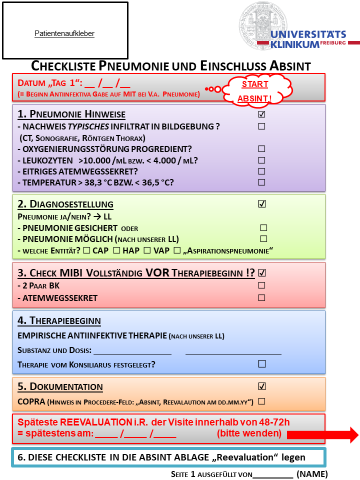


Figure 7. Graphical illustration of the checklist and reevaluation sheet applied in the intervention (in German).

## English translation of the checklist and reevaluation sheet:

CHECKLIST PNEUMONIA AND ABSINT INCLUSION

DATE: ___/___/___

(Only fill out this checklist if there is suspicion of pneumonia!)

1. PNEUMONIA INDICATIONS

- Evidence of a new infiltrate in imaging?

- Signs of infection present?

- Increased inflammatory parameters (CRP, PCT)?

- Leukocytosis (>10,000/μl or <4,000/μl)?

- Fever >38°C or hypothermia <36.5°C?

2. DIAGNOSTIC CLASSIFICATION

- Pneumonia (with lower respiratory tract infection)

- Possible pneumonia (no clear lower respiratory tract infection)

- Community-acquired pneumonia (CAP)

- Hospital-acquired pneumonia (HAP)

3. CHECK MICROBIOLOGY BEFORE STARTING THERAPY

- 2x blood cultures

- Sputum/expectorate collection

4. THERAPY DECISION

- Empirical antibiotic therapy (according to guidelines)

- Substance and dose: ____________

- Was a consultation required? ☐ Yes ☐ No

5. DOCUMENTATION

Documentation: Antibiotics in protocol-ABSINT (start, reevaluation, end)

Latest reevaluation within 48-72 hours during rounds!

REEVALUATION

48-72 hours after start of therapy, Date: ___/___/___

Is the diagnosis of pneumonia confirmed?

**✅ Pneumonia confirmed or possible**

1. Pathogen detected?

- Yes, in respiratory secretions: ____________

- Yes, in blood culture: ____________

- No ☐

2. Adjustment to targeted therapy?

- Yes, adapted to pathogen (substance and dose): ____________

- Yes, according to consultation recommendation

- No, because:

- No clear diagnosis yet

- Pathogen not identified

- Other reason: ____________

3. Planned therapy duration?

- Yes, planned for: ___ days

- No, reason: ____________

**No Pneumonia**

- ☐ Differential diagnosis: ____________

- Antibiotic therapy discontinued?

- Yes, due to:

- Alternative infection diagnosis

- No infection

- Other reason: ____________

- No, reason: ____________

DOCUMENTATION

- Recorded in the patient file

- Treatment team and attending physician informed

- Therapy decision shared in team rounds

📝 This checklist should be filed in the ABSINT folder under “Reevaluation”

📂 After completion, place this checklist in the “Completed” folder

# Local Guideline

**MANAGEMENT OF PNEUMONIA IN ICU PATIENTS**

GUIDELINES FOR MEDICAL INTENSIVE CARE (MIT 1 & 2)

**Contents**

1. Objectives p.2
2. Definitions p.3
3. Pathogens p.4
4. Diagnostics p.5
5. Criteria for Confirming Pneumonia Diagnosis p.7
6. Therapy p.8
   6.1. Principles p.8
   6.2. Initial Empirical Therapy p.9
7. Critical Reevaluation p.12
8. Targeted Therapy p.13
9. Parapneumonic Effusion p.14
10. VAP Prevention p.14
11. MIT-ABSINT p.14
12. Documentation MIT-ABSINT p.15
13. Guideline Group p.16

**1. Objectives of the Guidelines**

**Background**

**Diagnostic Confirmation**:

Diagnosing pneumonia in ICU patients is not straightforward—especially in intubated and mechanically ventilated patients. Supine chest X-rays rarely appear normal, oxygen supplementation is almost always required, and inflammatory markers are rarely within the normal range. However, these clinical parameters are not specific to pneumonia. Non-infectious differential diagnoses—such as acute heart failure with pulmonary edema—must also be considered.

**Diagnostics**:

Adequate diagnostics not only improve diagnostic accuracy but also enable guideline-conform and pathogen-specific antibiotic therapy.

**Empirical Therapy**:

Prompt initiation of adequate empirical antibiotic therapy is necessary and prognostically significant for these critically ill patients.

**Current Deficiencies:**

In suspected pneumonia cases, diagnostics are often incomplete. Especially a comprehensive microbiological diagnostics before starting antibiotics is missing, making it impossible to switch to targeted therapy in the later course. Additionally, once all results are available, the initial presumptive diagnosis is often not routinely reevaluated, leading to prolonged empirical therapy. This behaviour increases antibiotic consumption—especially of broad-spectrum agents—in ICU wards.

**Aims of the Guideline:**

1. Completion of essential diagnostics before starting empirical antibiotic therapy
2. Timely initiation of adequate empirical therapy following proper diagnostics
3. Critical reevaluation of empirical therapy (after 3 days)
4. Switch to targeted antibiotic therapy using the most effective agent for the identified pathogen (“S” on antibiogram does not always mean “best therapy”)
5. Discontinuation of empirical therapy when an alternative diagnosis is confirmed (e.g., acute heart failure with pulmonary edema instead of pneumonia)
6. Definition of appropriate treatment duration

**2. Definitions**

**Types of Pneumonia**

- CAP (Community-acquired pneumonia): Acquired outside the hospital in immunocompetent patients
- HAP (Hospital-acquired pneumonia): Occurs > 48h after hospitalization
  - VAP (Ventilator-associated pneumonia):

Occurs > 48h after endotracheal intubation

- Pneumonia in Immunosuppression (either community- or hospital-acquired):
  - Neutropenia (<1000/µL neutrophils)
  - Long-term immunosuppressive medication (e.g., systemic steroids >10 mg/day for >4 weeks)
  - Solid organ transplantation
  - Stem cell transplantation
  - HIV with low CD4 count (<200/µL) or AIDS
  - Antibody deficiency syndromes
  - Congenital immune disorders

**Severity Levels of Pneumonia**

- Non-severe pneumonia: No relevant oxygenation disorder
- Severe pneumonia: Presence of a clinically relevant oxygenation disorder
  - Defined as acute and progressive and not fully explained by other diagnoses

Risk Factors for Infection with:

| **MRSA** | **Pseudomonas aeruginosa** | **MRGN (ESBL+ Enterobacteriaceae)** |
| --- | --- | --- |
| Known MRSA colonization | COPD Gold IV | Known colonization (in last 3 months) |
| Prior MRSA infection | Cystic fibrosis | Recent broad-spectrum antibiotic use |
|  | Bronchiectasis |  |
|  | Known colonization |  |

**Note:** Risk classification is based on both scientific evidence and local resistance patterns. This table is critical for choosing the correct empirical therapy.

**3. Pathogen Spectrum**

| **Pathogens in severe CAP** | **Frequency (in percent)** |
| --- | --- |
| Streptococcus pneumoniae | 30% |
| Staphylococcus aureus (MSSA) | 10% |
| Viruses (esp. Influenza from November until March) | 20% |
| Legionella spp. | 10% |
| Enterobacteriaceae (E. coli, Klebsiella) | 10% |
| Haemophilus influenzae | 5% |
| Mycoplasma pneumoniae | 5% |
| Pseudomonas aeruginosa | <5% |
| Rare CAP pathogens: C. burnetii, C. psittaci, F. tularensis, M. tuberculosis |  |

**Pathogens in HAP and VAP**
Often broad-spectrum, sometimes polymicrobial. S. aureus often leads as a single pathogen, but gram-negative bacteria are more frequent overall.

| **Pathogen** | **Frequency in percent** |
| --- | --- |
| Staphylococcus aureus | ~25% |
| Enterobacteriaceae (E. coli, Klebsiella, Enterobacter) | ~25% |
| Pseudomonas aeruginosa | 10% |
| Streptococcus pneumoniae | 10% |
| Haemophilus influenzae | <10% |
| Acinetobacter spp. | <10% |
| Stenotrophomonas maltophilia | <10% |

- Assess patient’s clinical picture to differentiate between colonization and true infection
- In long-term ventilation (> 5 days): more Pseudomonas and Stenotrophomonas, fewer pneumococci
- Rare HAP/VAP pathogens: Legionella spp., Aspergillus spp., seasonal viruses (e.g. influenza)
- Non-relevant pathogens (commensals): Candida spp., viridans streptococci, coagulase-negative staphylococci, Enterococcus spp., Corynebacterium spp., Neisseria spp. (except rare N. meningitidis), non-influenza Haemophilus spp.

**4. Diagnostics**

**General Measures**

- Medical history (e.g., prior splenectomy, vaccination), physical exam, labs, ABG, temperature

**Imaging**

- Aim to confirm typical pulmonary infiltrates
- Supine chest X-ray often inadequate for ICU patients
  ➤ CT scan generally required
  ➤ Critically review the radiology report
- Ultrasound: can reliably detect infiltrates and help rule out differential diagnoses (e.g., B-lines = pulm. edema)
- Pleural effusion: Perform thoracentesis if present (see Section 9)

**Blood Cultures**

- 2 pairs before starting antibiotics, even if prior ABx were given

**Respiratory Secretions**

- Involve nursing team for timely sampling
- Always collect before antibiotics
- Non-intubated patients: sputum only with productive cough; may do 1-time tracheal suction
- Intubated patients: tracheal aspirate and BAL are equivalent
  - Exception: suspected invasive pulmonary aspergillosis ➤ BAL targeted to previous CT finding
  - Caution: BAL in suspected TB or abscess only in exceptional cases due to risk of dissemination

**Mandatory diagnostics before starting therapy**

- Imaging
- Blood cultures
- Respiratory secretions

Caution in shock: Do not delay therapy for diagnostics.
Goal: Diagnostics + therapy initiation within 1 hour

In (suspected) pneumonia without shock: Delay of up to 8h is acceptable before starting antibiotics

**Additional Measures**

- Legionella Ag in urine: CAP + severe HAP
- Multiplex PCR from BAL or (nasopharyngeal swab alternative) for severe CAP in immunosuppressed or CF
- Influenza rapid test (Nov–Mar): always in season

**Special Case: Severe Immunosuppression**

- Broader diagnostic scope (empirical therapy remains unchanged)
- CT thorax mandatory (low-dose, no contrast OK)
- Consider early targeted BAL (e.g., in Aspergillus-suspicious infiltrates)
- Microbiology from BAL:
  - Multiplex PCR (BAL preferred, nasal swab if not possible)
  - Tests for: bacteria incl. mycobacteria and nocardia, fungi (culture + PCR), Aspergillus Ag, Pneumocystis

**Aspergillus Pneumonia**

- Diagnosis is difficult; imaging and antigen tests may be false positives
- Empirical therapy justifiable in typical infiltrates, severe immunosuppression, and severe disease
- Otherwise: require typical infiltrates and positive BAL antigen and/or Aspergillus from culture/PCR
- Additional: serum Aspergillus antigen + beta-D-glucan to improve certainty

Caution: Tracheal Aspergillus Ag often false positive

**On ICU Admission with prior Empirical Treatment**

- Always draw 2 sets of blood cultures, even to prove absence of bacteremia/fungemia
- Collect respiratory samples (or repeat)
- Review imaging
- Reassess pneumonia suspicion

**5. Criteria for Confirming Pneumonia Diagnosis**

Diagnosing pneumonia is challenging. No symptom is specific, so a combination of clinical criteria is required.

| **Confirmed Pneumonia** | **Possible Pneumonia** |
| --- | --- |
| New/progressive infiltrate and at least 2 of the following: | New/progressive infiltrate and at least 1 of the following: |
| - Fever/hypothermia | - Fever/hypothermia |
| - WBC >10,000 or <4,000 /µl | - WBC >10,000 or <4,000 /µl |
| - Purulent secretions | - Purulent secretions |
| - Oxygenation disorder | - Oxygenation disorder |
| - Typical pathogen | - Typical pathogen |

Do not start therapy based solely on elevated inflammatory markers!

**6. Therapy**

**6.1. Principles**

**Penicillin Allergy:**
Needs critical review—only ~10% of reported allergies are real. Only anaphylaxis matters, not rash or diarrhea. Testing recommended. If true allergy: use Meropenem. Caution: In case of prior anaphylaxis to penicillin, cross-reactivity with Meropenem ≈ 0.5%

If Empirical Therapy Already Started Before ICU Admission:
If therapy is inadequate (wrong diagnosis or not guideline-conform), it should be discontinued or adjusted upon ICU admission.

**Special Case - Septic Shock**
Add high-dose single dose of Tobramycin (6–7 mg/kg, max 600 mg IV over 1h) to standard therapy.

Goal: Maximize bactericidal effect while minimizing toxicity before pathogen identification

**Duration of Therapy:**

- Mild CAP: 5 days (if improved)
- Severe CAP/HAP/VAP: 7 days (if improved)
- Clarithromycin: 3 days only in severe CAP (even if Legionella Ag negative). Not used in mild CAP!
- S. aureus bacteremic pneumonia: 4 weeks (after Infectious Disease consultation)
- Invasive Aspergillus pneumonia: ≥6 weeks (after Infectious Disease consultation)

**Special Case: Severe Immunosuppression**
No change to empirical therapy—pneumococci remain most frequent pathogens.

**Suspected Aspergillus Pneumonia**
Empirical therapy only in severe immunosuppression and severe pneumonia:

- 1st choice: Voriconazole (Day 1: 2 × 6mg/kg IV over 1h; from Day 2: 2 × 4mg/kg IV over 1h)

Caution: Side effects, liver toxicity, interactions

- If liver disease: Caspofungin as alternative (70 mg IV Day 1, then 50 mg IV daily)

**Inhaled Antibiotics**
No robust data—case-by-case decision after ID and pulmonology consult

**ID Consultation Recommended for:**

- MDR pathogen-confirmed CAP/HAP/VAP
- Therapy ≥5 days with no improvement
- 4MRGN detection (Carbapenem-resistant)
- TB or NTM detection
- Aspergillus pneumonia

**6.2. Initial Empirical Therapy in the ICU**

**Dosing Clarification:**

- No shock: Dosage listed = initial dose; adjust based on renal function from 2nd dose onward
- Shock**:** Dosage listed = for first 24h; adjust from Day 2 based on renal function

**Note:**
If previous pathogens (last 3 months) with resistance profiles exist, use that information to guide empirical therapy.
